# Frequency and severity of systemic reactions during beta-lactam skin testing in adults with immediate hypersensitivity allergy

**DOI:** 10.1186/s13223-026-01032-2

**Published:** 2026-04-15

**Authors:** Patricia Letón-Cabanillas, Blanca Noguerado-Mellado, Patricia Quijada-Morales, Gema Salas-Parra, Patricia Rojas-Pérez-Ezquerra

**Affiliations:** 1 Allergy Department, Hospital General Universitario Gregorio Marañón, 46Th Dr Esquerdo St., 28007 Madrid, Spain; 2 Gregorio Marañón Health Research Institute (IiSGM), Madrid, Spain

**Keywords:** Immediate hypersensitivity, Beta-lactams, Drug allergy, Penicillin allergy, Skin testing, Systemic reactions, Intradermal test

## Abstract

Hypersensitivity to beta-lactams (BL) is the most frequent drug allergy, and skin testing (ST) remains the first-line diagnostic tool. Although generally safe, systemic reactions (SR) during ST are a concern. We conducted a 7-year ambispective study (2018–2025) including 216 adults with confirmed immediate hypersensitivity reactions (HSR) to BL, established by positive skin tests (ST) or drug challenge tests (DCT). Among them, 138 (63.9%) had positive ST, predominantly intradermal tests (IDT; 93.5%). Five patients (3.6% of ST-positive; 2.3% of the entire cohort) developed SR during ST, all after IDT following negative skin prick tests (SPT). Reactions were mostly mild (urticaria, generalized pruritus, erythema), although one anaphylaxis occurred. All were rapidly controlled with symptomatic treatment. Surprisingly, all patients with SR had urticaria–angioedema as their index reaction; none had experienced prior anaphylaxis. A significant shorter time interval between the index reaction and allergy evaluation was observed in SR patients (mean 23.6 vs. 48.8 months, *p* = 0.02). No significant associations were identified for age, sex, culprit BL, or specific IgE. SR during BL ST are infrequent but clinically relevant. SPT appears highly safe, whereas IDT requires particular caution, especially when performed shortly after the index reaction.

## Introduction

Hypersensitivity to beta-lactams (BL), particularly to the aminopenicillin subgroup, is one of the most important and common drug allergies [1].

The first step in the diagnosis of immediate hypersensitivity reactions (HSR) to BL is skin testing (ST), which is generally considered safe, especially when performed sequentially with a skin prick test (SPT) and intradermal test (IDT) with readings at 15 min, and when the proper concentrations are used. However, the risk of systemic reactions (SR) during the ST exists [2–8] and must be considered. Most of these reactions occur in patients with immediate HSR.

This study aimed to analyze the frequency and severity of these SR with BL positive ST in adult patients with immediate HSR to BL.

## Methods

An ambispective study was conducted over a 7-year period (July 2018-June 2025). Data from July 2018 to December 2023 were collected retrospectively and from January 2024 to June 2025 prospectively, using identical diagnostic procedures throughout, involving 216 adult patients (mean age 62 (SD 15.9), 67.1% women) with a confirmed immediate HSR to BL by positive ST or positive drug challenge tests (DCT) (Table 1).

**Table 1:** Clinical characteristics of patients diagnosed with positive skin tests (ST) versus with positive drug challenge test (DCT)

	Positive ST	Positive DCT
**n (%)**	138 (63.9%)	78 (36.1%)
**Age [mean (SD)]**	61 (16.2) years	62 (16.9) years
**Sex**^a^** [n (%)]**	F 96 (69.6%)	F 52 (66.7%)
***Index reaction *[n (%)]**		
Anaphylaxis	62 (44.9%)	31 (39.7%)
U-AE^b^	76 (55.1%)	47 (60.3%)
***BL***^c^*** responsible* [n (%)]**		
Penicillin	12 (8.7%)	3 (3.8%)
Aminopenicillins	62 (44.9%)	44 (56.4%)
Clavulanic acid	7 (5.1%)	2 (2.6%)
Cephalosporins	35 (25.4%)	21 (26.9%)
Piperacillin-tazobactam	2 (1.4%)	7 (9%)
Meropenem	2 (1.4%)	0 (0%)
BL ring^c^	18 (13.1%)	1 (1.3%)

^a^Female (F)

^b^Urticaria-angioedema (U-AE)

^c^Beta lactam (BL) ring: This group included patients sensitized to both amoxicillin and penicillin, consistent with beta-lactam ring hypersensitivity rather than isolated side-chain sensitization

**Fig. 1:**
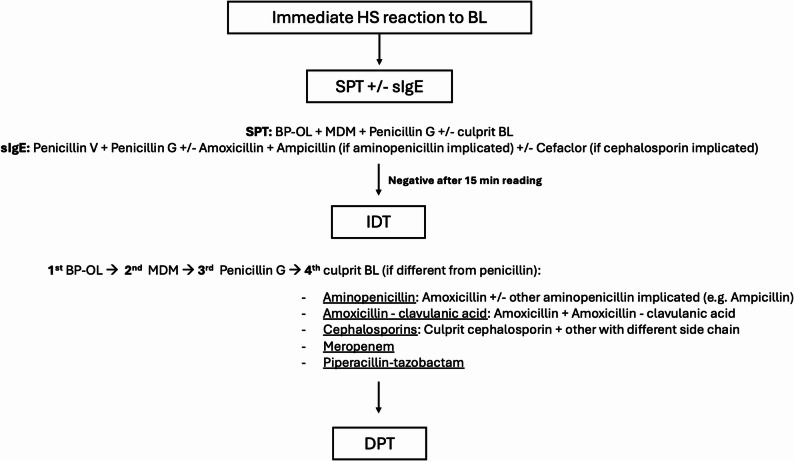
Diagnostic workflow for beta-lactam immediate hypersensitivity skin testing

The allergy workout included ST with benzylpenicilloyl polylysine extract (Benzylpenicilloyl-octa-L-lysine: BP-OL, 0.04 mg/mL, DAP®, Diater, Leganés), minor determinant mixture extract (Sodium benzylpenilloate: MDM, 0.5 mg/mL, DAP®), freshly prepared penicillin G, the culprit BL and selected alternative BL based on clinical history. The same drug concentrations and extracts were used for both SPT and IDT (Table 2). SPT were performed first with all selected reagents and read after 15 min. SPT were considered positive when the wheal diameter was ≥ 3 mm compared with the negative control [1]. Only in the case of negative SPT results, intradermal tests (IDT) were initiated. IDTs were performed sequentially, administering one drug at a time, with a 15-min interval between each test to allow for immediate reading before proceeding to the next. The testing order was BP-OL, MDM, penicillin G, followed by the suspected BL and/or alternative related molecules when clinically indicated. For aminopenicillins, amoxicillin was always tested, and the suspected aminopenicillin was also tested when different. In cases where amoxicillin–clavulanic acid was the culprit drug, both amoxicillin and amoxicillin–clavulanic acid were tested. For cephalosporins, the culprit cephalosporin and an additional cephalosporin from a different structural group were evaluated [2]. This diagnostic workflow is summarized in Fig. 1.

**Table 2:** Concentrations for beta-lactams skin tests

Betalactam	Skin prick and intradermal test
BP-OL extract	6 × 10^–5^ M
MDM extract	0.5 mg/mL
Penicillin G (Benzylpenicillin)	10.000 units/mL
Amoxicillin	20 mg/mL
Amoxicillin–clavulanic acid	20 mg/mL
Ampicillin	20 mg/mL
Cefuroxime	20 mg/mL
Ceftriaxone	20 mg/mL
Cefotaxime	2 mg/mL
Ceftazidime	2 mg/mL
Cefepime	2 mg/mL
Meropenem	2 mg/mL
Piperacillin-tazobactam	20 mg/mL

^*^BP-OL (Benzylpenicilloyl polylysine), MDM (Minor determinant mixture)

High-risk patients (previous anaphylaxis) underwent IDT with stepwise dilutions (to 10⁻^3^), while low-risk patients were tested directly with validated non-irritant concentrations. Patient´s age, sex, index reaction history, time interval from reaction to assessment, past medical history, culprit BL and allergy workout were retrieved from medical records.

DCTs were performed using a two-step protocol, consisting of administration of 25% of the therapeutic dose followed by the remaining 75% after a 30-min interval if no reaction occurred. Patients were monitored for at least 2 h after the final dose [1].

In patients whose index reaction had occurred more than 1 year before evaluation and who had an initially negative allergy work-up (ST and DCT), ST was repeated (“retest”) between 1 and 6 months after the first assessment [1], following the same protocol.

In several cases, specific immunoglobulin E (sIgE) to amoxicillin, ampicillin, cefaclor, penicilloyl V and penicilloyl G (Thermo Fisher Scientific®, United Kingdom) was measured (positive value ≥ 0.35 kUA/L).

Statistical analysis was performed using the Student’s T-test for quantitative and Fisher´s exact test for nominal categorical variables, values of *p* ≤ 0.05 were considered statistically significant.

The ethical committee of our hospital gave ethical clearance. All patients signed written informed consent for drug allergy testing.

## Results

From the 216 patients with immediate HSR, 138 (63.9%) had positive ST. The mean age in this group was 61 years (SD 16.2) and 69.6% were women. Among them, 9 (6.5%) had positive SPT, with 6 (67%) having anaphylaxis as the index reaction and 3 (33%) having urticaria-angioedema (U-AE). The remaining 129 (93.5%) had positive IDT, of which 56 (43%) had anaphylaxis as the index reaction and 73 (57%) had U-AE. The mean time elapsed from reaction to test was 47.9 months (SD 117.8). The BL implicated were amoxicillin (43.5%), cephalosporins (25.4%), amoxicillin+penicillin (BL ring; 14.5%), penicillin (8.7%), clavulanic acid (5.1%), piperacillin–tazobactam (1.4%) and meropenem (1.4%).

Five (3.6%) patients experienced SR during ST (Table 3), all of which occurred after IDT, with previous negative SPT. Four patients had a negative value in sIgE, not performed for the fifth. Amoxicillin was the causative agent in 3 cases, while MDM and cefuroxime were responsible for 1 case each. Most of the reactions were mild [3] (4 patients had generalized pruritus, erythema and/or urticaria, and 1 patient had lingual angioedema) except for 1 patient that had an anaphylaxis. The onset of the severe reaction was earlier (10 min after IDT), while mild reactions had an onset within 15–20 min. They were treated with antihistamines (dexchlorpheniramine 5 mg subcutaneously), and in the 1 case that experienced an anaphylaxis, 0.3 mg of intramuscular epinephrine and methylprednisolone 60 mg was administered with complete remission. First-generation antihistamines were administered as they were the available intravenous option in our center.

**Table 3:** Characteristics of patients with systemic reactions following skin testing

			Index reaction					Systemic reaction with IDT		
	Age	Sex^a^	Drug^b^	Symptoms	Reaction-test interval^c^	sIgE^d^ (kU_A_/L)) Positive ≥ 0.35	+ IDT^e^	Symptoms (brown grade)	Treatment^f^	Onset
1	67	F	AMC	Generalized urticaria	15 months	PNG 0 PNV 0.02 AMX 0.11	PNG, **AMX**	Localized urticaria (mild)	AH	15 min
2	62	F	AMC	Facial erythema, palmo-plantar pruritus	24 months	PNG 0 PNV 0.06 AMX 0.08	**AMX**	Generalized pruritus (mild)	AH	20 min
3	56	F	AMC	Generalized pruritus, nausea	10 months	Not performed	PNG, **MDM**	Facial erythema, palmo-plantar pruritus (mild)	AH	15 min
4	68	F	AMP	Palmo-plantar pruritus	5 years	PNG 0.05 PNV 0.06 AMX 0.3	**AMX retest**	Thoracic erythema, genital pruritus (mild)	AH	20 min
5	78	F	CFT	Generalized urticaria	3 months	PNG 0.04 PNV 0.05 AMX 0.1 AMP 0.09 CFO 0.05	PNG, **CFX**	Generalized urticaria, nausea, and syncope (severe)	AH + CC + E	10 min

^a^Female (F)

^b^Amoxicillin (AMX), Amoxicillin–clavulanic acid (AMC), Ampicillin (AMP), Ceftriaxone (CFT), Cefuroxime (CFX)

^c^Time elapsed from index reaction to test

^d^Specific IgE (sIgE), Penicilloyl G (PNG), Penicilloyl V (PNV), Amoxicillin (AMX), Ampicillin (AMP), Cefaclor (CFO). Positivity cut-off ≥ 0.35 kU_A_/L

^e^Only shown IDT with positive result. Previous SPT were negative. IDT with systemic reaction in bold: because IDTs were performed sequentially at 15-min intervals, the reagent administered immediately before symptom onset was considered the most likely trigger. In Patient 5, the IDT to cefuroxime was performed first and triggered the systemic reaction; therefore, the IDT to ceftriaxone was not performed

^f^Antihistamine (AH), Corticosteroid (CC), Epinephrine (E)

The SR rate for all patients with a diagnosis of immediate HSR (n = 216) was 2.3%, and 3.6% for the patients that had a positive skin test (n = 138).

In all patients (100%) with SR after ST, the index reaction was U-AE (Table 3). None had a history of anaphylaxis. The mean time from index reaction to ST was 23.6 months (SD 24.9) for SR patients vs. 48.8 months (SD 119.8) for non-SR (*p* = 0.02, Student’s T-test), a 50% reduction in time for the SR group (Table 4). Aminopenicillin was the most frequent BL in SR (60%), though not statistically significant (*p* = 0.703, Fisher’s Exact Test), likely due to its high prevalence as culprit of the index reaction. No significant differences were found in age or sex.

**Table 4:** Comparison between patients with and without systemic reactions (SR) with positive beta lactam skin tests

	Without SR	With SR	*p value*
**n (%)**	133 (96.4%)	5 (3.6%)	
**Age [mean (SD)]**	61 (16) years	66 (8) years	*p* = *0.246*^+^
**Sex**^a^** [n (%)]**	F 95 (71.4%)	F 5 (100%)	*p* = *0.322*^*#*^
***Index reaction* [n (%)]**			
Anaphylaxis	62 (46.6%)	0	
U-AE^b^	71 (53.4%)	5 (100%)	*p* = *0.064*^*#*^
***BL***^c^*** responsible* [n (%)]**			
Penicillin	12 (9.2%)		
Aminopenicillins	58 (42.5%)	3 (60%)	*p* = *0.703*^*#*^
Clavulanic acid	7 (5.4%)		
Cephalosporins	34 (25.9%)	1 (20%)	*p* > *0.99*^*#*^
Piperacillin-tazobactam	2 (1.6%)		
Meropenem	2 (1.6%)		
BL ring^c^	17 (13.8%)	1 (20%)	*p* = *0.527*^*#*^
**Mean time elapsed from index reaction to test [mean (SD)]**	48.8 (119.8) months	23.6 (24.9) months	*p* = *0.02*^+^

^a^Female (F)

^b^Urticaria-angioedema (U-AE)

^c^Beta lactam (BL)

^#^Fisher’s exact test

^+^Student’s T-test

## Discussion

The frequency of SR after BL ST observed in our study aligns with rates reported in previous research, ranging from 0.1 to 2% in all tested patients and from 0.4 to 13.2% in patients with positive ST [4–8]. Despite reports of fatalities in the literature [9], most of the reactions in our study were mild except for one, and all had rapid response to symptomatic treatment.

Many of the available studies about SR after ST include mixed drug classes, combine immediate and delayed reactions, or involve paediatric populations in which systemic reactions are extremely rare [4–10], which limits comparability with adult cohorts such as ours. Co Minh et al., reported SR in 8.8% of patients with positive ST, half of these reactions occurred during SPT, 77% of those who reacted had a previous history of anaphylaxis, and also experienced anaphylaxis with ST [7]. Antico et al. observed reactions in 13.2% of positive ST, with 20% occurring during SPT and amoxicilin was the culprit drug in 80% of cases. Among these, 60% had a previous history of anaphylaxis, and 20% experienced anaphylaxis after skin testing [4]. More recent studies have shown varying results. Luliano et al. [10], reported SR in 1 patient that developed generalized urticaria following both SPT and IDT to amoxicillin/clavulanate. Karavaizoglu et al. [8], observed SR in 0.4% of children tested, 2 reactions after SPT and 2 after IDT.

We observed that a shorter time interval between the index reaction and ST might increase the risk of SR. However, it is equally important to avoid prolonged intervals, as such late testing may result in a loss of skin reactivity, potentially compromising diagnostic accuracy [1]. To the best of our knowledge, this association has not been previously described in the literature. Given the small number of SR (n = 5), however, this association should be interpreted cautiously and considered hypothesis-generating. Previously identified risk factors for SR, such as severity of index reaction, culprit BL, positive sIgE or SPT, gender, or age, were not applicable to our study population.

Several methodological aspects should also be considered. The cephalosporin concentrations used in our protocol were lower than the currently recommended maximum non-irritant concentrations (20 mg/mL). Regarding amoxicillin, although higher concentrations for skin prick testing (up to 200 mg/mL) have been explored in some studies and may increase sensitivity, they are not currently standardized. Therefore, the sensitivity of skin testing in our cohort may have been underestimated.

In addition, a relatively high proportion of patients with confirmed immediate hypersensitivity had negative skin tests but positive drug challenge tests. This may be explained by factors such as loss of IgE sensitization over time, selective sensitization not fully captured by classical determinants, or the inclusion of patients with a high clinical suspicion of allergy. Therefore, negative skin tests should be interpreted with caution, particularly in patients with a convincing clinical history.

## Conclusion

In conclusion, a shorter interval between the index reaction and testing showed a possible association with SR; however, no firm conclusions can be drawn given the small number of cases, and this finding requires confirmation in further larger studies. No other strong predictors of SR were identified in our cohort. In our experience, SPTs were safe with no systemic reactions. Aminopenicillins showed a higher, though non-significant, SR incidence, likely due to their frequent implication. SR during BL skin testing are not negligible, so healthcare providers, especially for IDT, should be prepared to manage and treat SR effectively.

## Data Availability

No datasets were generated or analysed during the current study.

## Abbreviations

BL: Beta-lactams
HSR: Hypersensitivity reactions
ST: Skin testing
SPT: Skin prick test
IDT: Intradermal test
SR: Systemic reactions
DCT: Drug challenge test
sIgE: Specific immunoglobulin E
U-AE: Urticaria–angioedema
MDM: Minor determinant mixture
SD: Standard deviation
BP-OL: Benzylpenicilloyl-octa-L-lysine
F: Female
AMX: Amoxicillin
AMC: Amoxicillin clavulanic acid
AMP: Ampicillin
CFT: Ceftriaxone
CFX: Cefuroxime
PNG: Penicilloyl G
PNV: Penicilloyl V
CFO: Cefaclor
AH: Antihistamine
CC: Corticosteroid
E: Epinephrine
